# Skipping the Insect Vector: Plant Stolon Transmission of the Phytopathogen ‘*Ca.* Phlomobacter fragariae’ from the *Arsenophonus* Clade of Insect Endosymbionts

**DOI:** 10.3390/insects12020093

**Published:** 2021-01-22

**Authors:** Jessica Dittmer, Thierry Lusseau, Xavier Foissac, Franco Faoro

**Affiliations:** 1 Dipartimento di Scienze Agrarie e Ambientali (DISAA), Università degli Studi di Milano, Via Celoria 2, 20133 Milano, Italy; franco.faoro@unimi.it; 2 Faculty of Science and Technology, Free University of Bozen-Bolzano, Piazza Università 5, 39100 Bolzano, Italy; 3 Université de Bordeaux, INRAe, Biologie du Fruit et Pathologie, UMR1332, 71 avenue Edouard Bourlaux, CS20032, 33882 Villenave d’Ornon, France; thierry.lusseau@inrae.fr (T.L.); xavier.foissac@inrae.fr (X.F.)

**Keywords:** Strawberry Marginal Chlorosis, plant-microbe interactions, pathogen transmission, planthopper

## Abstract

**Simple Summary:**

Numerous plant sap-feeding insects are vectors of plant-pathogenic bacteria that cause devastating crop diseases. Some of these bacteria had initially been insect endosymbionts that eventually evolved the capacity to survive in plants after being frequently transmitted to plants by their insect hosts during feeding. An example for this evolutionary transition is the bacterial symbiont ‘*Candidatus* Phlomobacter fragariae’ (hereafter *Phlomobacter*) of the planthopper *Cixius wagneri.* Upon transmission to strawberry plants by its insect vector, the bacterium accumulates in the plant phloem and causes Strawberry Marginal Chlorosis disease. Using quantitative PCR and transmission electron microscopy, we demonstrate an additional plant-to-plant transmission route: *Phlomobacter* can be transmitted from an infected plant to daughter plants through stolons, a specific type of stem from which daughter plants can develop. Our results show that *Phlomobacter* was abundant in stolons and was efficiently transmitted to daughter plants, which developed disease symptoms. Hence, *Phlomobacter* is not only able to survive in plants, but can even be transmitted to new plant generations, independently from its ancestral insect host.

**Abstract:**

The genus *Arsenophonus* represents one of the most widespread clades of insect endosymbionts, including reproductive manipulators and bacteriocyte-associated primary endosymbionts. Two strains belonging to the *Arsenophonus* clade have been identified as insect-vectored plant pathogens of strawberry and sugar beet. The bacteria accumulate in the phloem of infected plants, ultimately causing leaf yellows and necrosis. These symbionts therefore represent excellent model systems to investigate the evolutionary transition from a purely insect-associated endosymbiont towards an insect-vectored phytopathogen. Using quantitative PCR and transmission electron microscopy, we demonstrate that ‘*Candidatus* Phlomobacter fragariae’, bacterial symbiont of the planthopper *Cixius wagneri* and the causative agent of Strawberry Marginal Chlorosis disease, can be transmitted from an infected strawberry plant to multiple daughter plants through stolons. Stolons are horizontally growing stems enabling the nutrient provisioning of daughter plants during their early growth phase. Our results show that *Phlomobacter* was abundant in the phloem sieve elements of stolons and was efficiently transmitted to daughter plants, which rapidly developed disease symptoms. From an evolutionary perspective, *Phlomobacter* is, therefore, not only able to survive within the plant after transmission by the insect vector, but can even be transmitted to new plant generations, independently from its ancestral insect host.

## 1. Introduction

Many insect species maintain long-lasting associations with heritable bacterial endosymbionts. Some of these bacteria provide important services to their hosts (e.g., essential nutrients lacking from the host’s diet [1,2,3,4] or protection against pathogens [5,6,7]), whereas others interfere with host reproduction to promote their own vertical transmission [8,9]. Both beneficial and parasitic symbionts have important impacts on host ecology and evolution, enabling the exploitation of novel food sources [10] or influencing reproductive systems [11]. After millions of years of host–symbiont co-evolution, these bacteria are specifically adapted to living inside their host’s cells and often can no longer survive on their own [12,13]. In addition, numerous insect species are vectors of plant-pathogenic bacteria that cause devastating crop diseases and important economic losses [14,15,16]. The insect vectors are often phloem or xylem-feeding hemipterans (e.g., leafhoppers, planthoppers or psyllids), which acquire and transmit the bacteria while feeding on plants. Hence, these insect-vectored phytopathogens have evolved adaptations to very different host environments (insect and plant cells) and are able to switch frequently between these contrasting environments. However, it remains challenging to investigate the evolutionary transition from a purely insect-associated symbiont to an insect-vectored plant pathogen, due to the scarcity of symbiotic bacteria at the early stages of this transition.

Such bacteria occur in the *Arsenophonus* clade of insect endosymbionts. The genus *Arsenophonus* (Gammaproteobacteria) represents one of the most widespread clades of insect endosymbionts, with at least 5% of insect species estimated to be infected [17]. Members of the clade establish highly diverse symbiotic interactions with their insect hosts, ranging from reproductive parasitism (male-killing) in *Nasonia* wasps [18,19,20,21] to bacteriocyte-associated obligate mutualists in blood-feeding louse and bat flies, which provide B vitamins lacking from the host’s diet [22,23,24,25,26]. The latter have highly reduced genomes, a hallmark of long-term host-symbiont co-evolution in obligate insect endosymbionts [12,13]. In addition, numerous plant sap-feeding hemipterans harbour *Arsenophonus* symbionts, in some cases even in the bacteriome [4,27,28,29,30,31], but the functional role of these associations remains unknown. Despite the fact that *Arsenophonus* are thus firmly established as insect endosymbionts, two different strains belonging to the clade, ‘*Candidatus* Phlomobacter fragariae’ (hereafter *Phlomobacter*) and ‘*Ca.* Arsenophonus phytopathogenicus’, have been identified as insect-vectored plant pathogens [32,33]. Specifically, *Phlomobacter* is the causative agent of Strawberry Marginal Chlorosis (SMC) in France [34,35,36] and Japan [37], whereas ‘*Ca.* A. phytopathogenicus’ causes the disease “basses richesses” (=low sugar content) of sugar beet in France [38,39] and SMC in Italy [40]. Both bacteria are vectored by planthoppers of the cixiidae family, *Cixius wagneri* in the case of *Phlomobacter* and *Pentastiridius leporinus* for ‘*Ca.* A. phytopathogenicus’ [34,41,42]. The bacteria accumulate in the phloem of infected plants, ultimately causing leaf yellows, necrosis and plant death [35,39]. Since both strains are closely related to other *Arsenophonus* endosymbionts of diverse insects [32,43], it is likely that these bacteria were initially insect endosymbionts which subsequently evolved a plant pathogenic lifestyle. Considering that both diseases first appeared about 40 years ago and to date have only been observed in restricted and disconnected localities (France, Italy and Japan), it can be assumed (a) that the switch from ancestral purely insect-associated endosymbionts to insect-vectored plant pathogens occurred very recently and (b) that it occurred independently multiple times within the *Arsenophonus* clade [32,44]. These symbionts therefore represent ideal model systems to investigate bacterial adaptations at the early stages of transition towards insect-vectored phytopathogens.

To date, very little is known about the interactions between these bacteria and their respective insect and plant hosts apart from the obvious disease symptoms. In particular, SMC remains a serious problem for strawberry producers in south-western France, as the disease incidence can be very high in fruit production fields [34] and diseased plants exhibit stunted growth, small leaves, deformed and low-quality fruits as well as root necrosis [35]. Herein, we report new observations regarding the interaction between *Phlomobacter* and strawberry plants following the infection of the plant by the insect vector. Specifically, we provide evidence that *Phlomobacter* can be propagated from an infected plant to daughter plants via stolons. Stolons, also referred to as runners, are particular types of stems which grow horizontally above the soil surface and develop new clonal plants from buds. The daughter plants are nourished by the parental plant through the stolons, until a suitable spot is found where the daughter plant can take root. Our results demonstrate that *Phlomobacter* is present in the phloem sieve elements of stolons and is efficiently transmitted to daughter plants, resulting in a new transmission route independent from its ancestral insect host.

## 2. Materials and Methods

### 2.1. Plant Material

**Figure 1:**
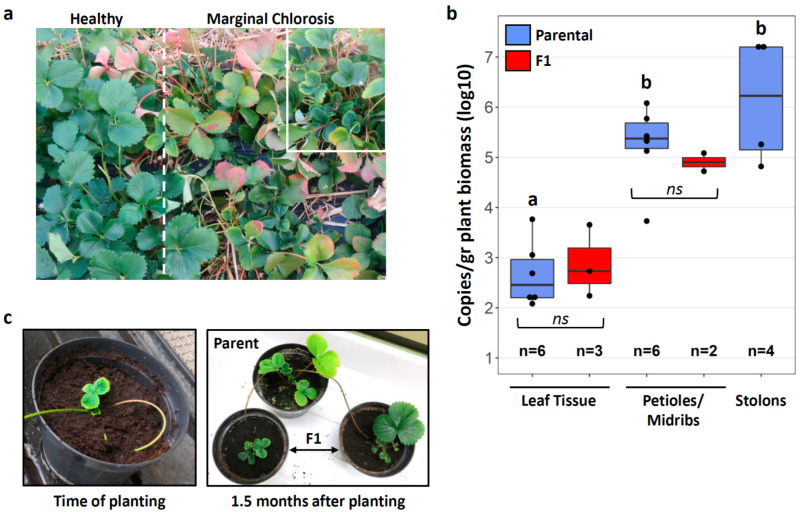
Stolon transmission of *Phlomobacter.* (**a**) Strawberry production field with both healthy and diseased plants, separated by the dashed line. The white box highlights plants showing the characteristic symptoms of Marginal Chlorosis diseases, i.e., small, cup-shaped leaves with yellow chlorosis along the leaf margins. (**b**) Quantification of *Phlomobacter* titers in different tissues of naturally infected parental plants (blue) and stolon-derived F1 daughter plants (red). Sample sizes are provided below each box plot. Different letters above the box plots indicate significant differences based on ANOVA followed by Tukey’s post-hoc test. (**c**) Leaves of stolon-derived daughter plants immediately developed disease symptoms.

A total of twenty strawberry plants (*Fragaria* × *Ananassa*, cultivar Cigaline) were collected in a strawberry production field in the Dordogne region (France) in October 2018. Only plants showing the characteristic symptoms of Marginal Chlorosis Disease, i.e., small, cup-shaped leaves with yellow chlorosis along the leaf margins ([35], Figure 1a), were uprooted, planted in individual pots and maintained in an environmental chamber at 22 °C, 55–60% relative humidity and a 14 h photoperiod. Almost a year later, in September 2019, two strawberry plants had developed stolons (runners) with daughter plants. The daughter plants (*n* = 5 from two parental plants) were planted in individual pots but remained connected to the parental plants through the stolons for 1–1.5 months.

### 2.2. DNA Extraction

DNA was extracted from six plants infected with *Phlomobacter* and collected in the field one year before, and three stolon-derived F1 daughter plants 1.5 months after planting. DNA was extracted from different parts of the plants, i.e., petioles and leaf midribs (*n* = 6 parental plants and 2 F1 plants), the remaining leaf tissue (*n* = 6 parental plants and 3 F1 plants), and stolons (*n* = 4, 2 from each parental plant). The effect size for the F1 plants was lower since some of these plants were still too small to obtain sufficient amounts of DNA, especially from petioles and leaf midribs. Total DNA was extracted from 0.5–0.9 g of plant biomass using the CTAB method: Plant material was ground in 4 mL CTAB buffer (2% CTAB (cetyltrimethylammonium bromide), 2% PVP K40, 1.4 M NaCl, 20 mM EDTA, 100 mM Tris-HCl, 0.02% β-mercaptoethanol) and incubated at 65 °C for 1.5 h. An amount of 750 µL of the homogenate were treated with 30 µg of RNase A at 37 °C for 30 min. Subsequently, the DNA was extracted twice with one volume chloroform/isoamyl alcohol (24:1 *v*/*v*) and precipitated in one volume of isopropanol after overnight incubation at −20 °C. The DNA pellet was resuspended in 30 µL of sterile water and DNA quality was verified using a NanoDrop spectrophotometer.

### 2.3. Quantification of Phlomobacter Titer in Plant Tissues

*Phlomobacter* titers were quantified using qPCR on a LightCycler LC480 (Roche). All samples were tested in duplicates with the previously published primers SpoT-F, SpoT-R and the FAM-labelled TaqMan probe SpoT-FAM-LNA [45] targeting the *spoT* gene of *Phlomobacter.* 20 µL reactions contained 1× Probes Master reaction mix (Roche), 0.5 µM of each primer, 0.25 µM of the SpoT-FAM-LNA probe, 5.5 µL of sterile water and 2 µL of template DNA. qPCR cycles consisted of an initial activation at 95 °C for 10 min, followed by 40 cycles of 95 °C for 15 s, 59 °C for 30 s and 72 °C for 30 s. *Phlomobacter* copy number/µL DNA extract was determined based on a standard curve obtained from the serial dilution of a purified *spoT* PCR product quantified using the Qubit dsDNA Broad Range Assay Kit (Invitrogen). *Phlomobacter* titers were normalized across all samples as *Phlomobacter* copy number/g plant biomass. Differences in *Phlomobacter* titer between plant tissues in parental plants were tested using one-way ANOVA followed by Tukey’s post-hoc test for multiple comparisons, since the data was normally distributed. Differences in *Phlomobacter* titer between parental and daughter plants were tested using Welsh’s *t*-tests. All statistical analyses were performed in R (R Project 3.6.3).

### 2.4. Transmission Electron Microscopy (TEM)

One month after planting of the F1 plants, two stolons (one from each parental plant) were removed from the plants and cut into 2 mm pieces. The cuttings were fixed overnight at 4 °C in 1 mL of sodium cacodylate buffer (Merck, Darmstadt, Germany) (0.1 M, pH 7.4) containing 4% paraformaldehyde and 4% glutaraldehyde. Subsequently, the plant cuttings were washed twice in sodium cacodylate buffer for 10 min and post-fixed overnight in 1% osmium tetroxide. Samples were dehydrated through serial ethanol washes (25–100%) and progressively infiltrated with Spurr’s resin, which was polymerized at 60 °C for 24 h. Semithin sections were stained with 0.1% toluidine blue (Merck, Darmstadt, Germany) and observed with an Olympus BX50 light microscope (Olympus, Waltham, MA, USA). Ultrathin sections were contrasted with 2% uranyl acetate and lead citrate and observed with a Jeol 100SX transmission electron microscope.

## 3. Results

### 3.1. Stolon Transmission of Phlomobacter

*Phlomobacter* titers were quantified in various tissues (petioles and leaf midribs, leaf tissue, stolons) of strawberry plants infected with the pathogen in the field. All of the tested plants were positive for *Phlomobacter* (Figure 1b), in accordance with their disease symptoms. It can be assumed that at the time of testing, the parental plants had been infected with *Phlomobacter* for at least 1.5 years. This is estimated based on the following considerations: The insect vector *C. wagneri* has two transmission periods in strawberry fields per year, one in spring and one in autumn [34,41]. In addition, symptoms appear two to three months after infection of the plant and the tested plants had already shown symptoms at the time of collection in autumn 2018. Therefore, it is likely that the tested plants had been infected with *Phlomobacter* by young *C. wagneri* adults in the spring of 2018, i.e., 1.5 years prior to testing in autumn 2019. In these stably infected plants, *Phlomobacter* titers were higher in petioles and leaf midribs than in the surrounding leaf tissue (Tukey’s post-hoc test: *p* = 0.00063), in accordance with its location in the phloem sieve tubes (Figure 1b). Average *Phlomobacter* titers in stolons were not significantly different from those in petioles and midribs (Tukey’s post-hoc test: *p* = 0.31). Nonetheless, pathogen titers in the stolons of the two tested plants differed by two orders of magnitude (Plant 1: 123,583 ± 57,820.64 bacteria/gr plant biomass; Plant 2: 15,991,747 ± 331,107.5 bacteria/gr plant biomass (mean ± SE)), indicating that the pathogen can reach exceptionally high abundances in stolons.

A total of five daughter plants developed from the stolons of these two plants, and their leaves immediately exhibited the characteristic symptoms of Marginal Chlorosis (Figure 1c). Accordingly, *Phlomobacter* titers measured in petioles/midribs and leaf tissue of 2–3 daughter plants 1.5 months after planting had already reached the same levels as in the parental plants (Petioles: Welsh’s *t*-test, *t* = −0.8963, *df* = 5.8354, *p* = 0.41; leaf tissue: *t* = 0.42005, *df* = 3.7245, *p* = 0.70) (Figure 1b). These observations indicate that the pathogen was efficiently transmitted from the parental plants to the daughter plants through the stolons.

### 3.2. Intracellular Localization of Phlomobacter in Stolons

**Figure 2:**
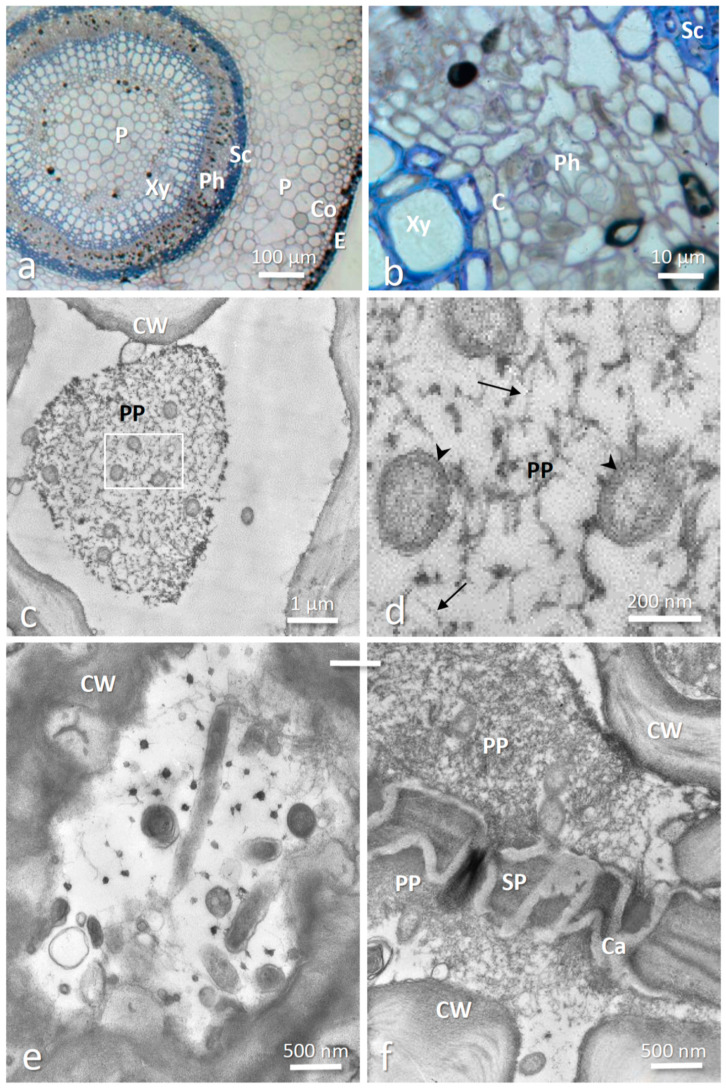
TEM observation of *Phlomobacter* in phloem sieve elements of stolons. (**a**,**b**) Cross-sections of an infected stolon stained with toluidine blue, showing the tissular organization and particularly the location of the phloem layer between the cambium and the sclerenchyma (**b**). (**c**–**f**) TEM micrographs of phloem sieve elements revealed the presence of numerous bacteria, which were frequently surrounded by P-protein filaments. (**d**) Enlarged view of the framed area in (**c**). The inner and outer membranes of the typical cell wall of Gram-negative bacteria are clearly visible (arrowheads), as well as the bead-string structure of P-protein filaments (arrows). (**e**) When cut longitudinally, the bacteria appeared as long rods, the typical shape of *Phlomobacter* and other bacteria of the genus *Arsenophonus.* (**f**) Bacteria and P-protein aggregates on both sides of a sieve plate lined by callose deposition between two sieve elements. C = Cambium; Ca = Callose; Co = Collenchyma; CW = Plant cell wall; E = Epidermis; P = Parenchyma; Ph = Phloem; PP = P-proteins; Sc = Sclerenchyma; SP = sieve plate; Xy = Xylem.

The anatomy of an infected stolon is presented in Figure 2a,b; showing the location of the phloem layer between the cambium and the sclerenchyma. TEM observations revealed numerous bacteria in mature phloem sieve elements (Figure 2c–f). The bacteria had the typical cell wall of gram-negative bacteria and were 0.2–0.3 µm in diameter and up to 2 µm long, although it is possible that some bacteria were longer because few were observed longitudinally (Figure 2e). These features correspond to previous descriptions of *Phlomobacter* in the phloem cells of strawberry petioles and leaf midribs [35,37] as well as ‘*Ca.* A. phytopathogenicus’ in the phloem cells of sugar beet and in diverse tissues of its planthopper vector [39,42]. The bacteria were generally distributed in the lumen of the phloem sieve elements and were frequently observed surrounded by dispersed P-protein filaments (Figure 2c,d). The latter had the typical appearance of filaments resembling strings of beads (Figure 2d) [46,47]. P-proteins are phloem-specific proteins that can rapidly plug the sieve pores to prevent pressure loss in neighboring cells in case of injury of the phloem sieve element [48,49,50] or in response to pathogen infection [51,52,53,54,55,56]. Accordingly, we also observed P-protein aggregates and bacteria on both sides of the sieve pores (Figure 2f).

## 4. Discussion

Our results demonstrate a plant-to-plant transmission route for *Phlomobacter*: Following the initial infection of a strawberry plant by the insect vector *C. wagneri*, the pathogen can be transmitted from this plant to clonal daughter plants through stolons. Combining quantitative PCR and transmission electron microscopy, we show that *Phlomobacter* can be present in the phloem sieve elements of stolons, reaching titers at least as high (if not higher) as in the petioles of the parental plant. Furthermore, the pathogen was efficiently transmitted to daughter plants and rapidly reached similar titers as in the parental plant, accompanied by the characteristic disease symptoms of Marginal Chlorosis. To our knowledge, this is the first demonstration of stolon transmission for a bacterial pathogen, whereas two fungal pathogens of strawberry, *Verticillium dahliae* and *Fusarium oxysporum* f. sp. *fragariae*, are known to be efficiently transmitted through stolons across multiple generations of stolon-derived plants [57,58]. Interestingly, the fungus-infected daughter plants were consistently free of disease symptoms, contrary to what we observed here for *Phlomobacter*.

An important line of defense against phloem-limited bacterial pathogens is the blocking of sieve elements to prevent pathogen spread through the phloem [51,52,53,54]. This is achieved through rapid plugging of the sieve pores by phloem-specific proteins (P-proteins), followed by callose deposition for permanent obstruction [46,47,48,49,50,55]. For instance, plugged sieve pores due to increased callose deposition typically accompany ‘*Ca.* Liberibacter asiaticus’ infections in citrus leaves [51,52]. Callose deposition and a change in P-protein conformation from a condensed (i.e., forisomes) to a dispersed state were also observed in broadbean leaves infected with Flavescence dorée Phytoplasma [53]. However, this physical barrier against the pathogen also reduces phloem transport of photoassimilates. Hence, plants have to face a trade-off between blocking pathogen spread and impairing nutrient flow. We hypothesize that the efficient pathogen transmission through stolons may be due to the fact that the parental plant cannot block its sieve elements in the stolons to prevent the spread of the bacterium, as this would also impair the nutrient provisioning of the daughter plant. Our transmission electron microscopy observations of *Phlomobacter* within the stolon sieve elements revealed that the bacterium was frequently surrounded by filamentous P-proteins in the lumen of the phloem cells. In addition, P-proteins were observed accumulating on both sides of the sieve plates, which may suggest a partial blocking of the sieve pores. However, the P-protein aggregates could also be an artefact from sample preparation, since the cutting of the stolons inevitably produced a depression in the phloem, which may have caused a blocking of the sieve pores before the tissue could be fully penetrated by the fixative. Therefore, it remains to be determined whether the accumulation of P-protein around the sieve pores was indeed due to the presence of *Phlomobacter*. In any case, it is unlikely that the nutrient flow was severely inhibited since the pathogen was transmitted to stolon-derived daughter plants. Alternatively, the defense response may have been triggered too late to prevent pathogen spread. Hence, investigating the impact of *Phlomobacter* infection on P-protein gene expression, P-protein conformation and nutrient flow in different plant tissues (e.g., petioles and stolons) will be an important step towards a better understanding of this disease.

Our observations may be of practical relevance for strawberry producers, as commercial strawberry plants are produced in nurseries by vegetative propagation from stolons. Therefore, stolon transmission of *Phlomobacter* could result in the production of infected plants in nurseries, unless symptoms develop quickly enough for the infected plants to be eliminated. A previous study revealed that *Phlomobacter* infection was rare in French nursery fields, despite a high disease incidence in fruit producing fields [34]. However, this may have been partly due to a spatial segregation between nurseries and the fruit producing regions (north-western vs. south-western France, respectively) and the picture may be different in nurseries established in areas with important insect vector populations. Considering that the data presented here is based on a small number of parental plants with long-term *Phlomobacter* infections, field-scale studies of stolon transmissions are necessary to obtain quantitative data on stolon transmission rate and disease incidence in daughter plants.

Furthermore, the stolon transmission of *Phlomobacter* is of interest in the context of the evolutionary transition from an ancestral insect endosymbiont towards an insect-vectored plant pathogen. Theory predicts that an initially insect-associated symbiont, which gets frequently transmitted to plants by its herbivorous insect host, may eventually evolve the capacity to survive in plants [15]. If this turns out to be detrimental for the plant, the former insect endosymbiont has become an insect-vectored phytopathogen. One might argue that *Phlomobacter* has gone one step further: Once transmitted to a strawberry plant by the insect vector, the bacterium is not only able to survive within the new plant host but could theoretically be transmitted to new plant generations through stolons, completely independently from its insect vector. This suggests a broad metabolic repertoire compared to other insect endosymbionts. Comparing the metabolic pathways encoded in the genome of *Phlomobacter* to those of other insect endosymbionts from the *Arsenophonus* clade will shed light on the genetic basis mediating this successful adaptation to the plant phloem sap habitat.

## 5. Conclusions

Plant pathogenic bacteria from the *Arsenophonus* clade are excellent study systems to investigate the evolutionary transition from insect endosymbionts to insect-vectored phytopathogens. The stolon transmission documented in this paper reveals that *Phlomobacter* is not only able to survive in strawberry plants but could even be transmitted to new plant hosts independently from its ancestral insect host. Future research is needed to better understand the potential bacterial adaptations mediating the switch from insect to plant hosts.

## Data Availability

The qPCR data presented in this study are available online as Supplementary Table S1.

## Supplementary Materials

The following are available online at https://www.mdpi.com/2075-4450/12/2/93/s1, Table S1: The qPCR data.

## References

[1] Nikoh N., Hosokawa T., Moriyama M., Oshima K., Hattori M., Fukatsu T. (2014). Evolutionary origin of insect-*Wolbachia* nutritional mutualism. Proc. Natl. Acad. Sci. USA.

[2] Hansen A.K., Moran N.A. (2011). Aphid genome expression reveals host-symbiont cooperation in the production of amino acids. Proc. Natl. Acad. Sci. USA.

[3] Koga R., Bennett G.M., Cryan J.R., Moran N.A. (2013). Evolutionary replacement of obligate symbionts in an ancient and diverse insect lineage. Environ. Microbiol..

[4] Santos-Garcia D., Juravel K., Freilich S., Zchori-Fein E., Latorre A., Moya A., Morin S., Silva F.J. (2018). To B or Not to B: Comparative Genomics Suggests *Arsenophonus* as a Source of B Vitamins in Whiteflies. Front. Microbiol..

[5] Oliver K.M., Russell J.A., Moran N.A., Hunter M.S. (2003). Facultative bacterial symbionts in aphids confer resistance to parasitic wasps. Proc. Natl. Acad. Sci. USA.

[6] Jaenike J., Unckless R., Cockburn S.N., Boelio L.M., Perlman S.J. (2010). Adaptation via symbiosis: Recent spread of a *Drosophila* defensive symbiont. Science.

[7] Chrostek E., Marialva M.S., Esteves S.S., Weinert L.A., Martinez J., Jiggins F.M., Teixeira L. (2013). *Wolbachia* variants induce differential protection to viruses in *Drosophila melanogaster*: A phenotypic and phylogenomic analysis. PLoS Genet..

[8] Werren J.H., Baldo L., Clark M.E. (2008). *Wolbachia*: Master manipulators of invertebrate biology. Nat. Rev. Microbiol..

[9] Hurst G.D., Frost C.L. (2015). Reproductive Parasitism: Maternally Inherited Symbionts in a Biparental World. Cold Spring Harb. Perspect. Biol..

[10] Ferrari J., Darby A.C., Daniell T.J., Godfray H.C.J., Douglas A.E. (2004). Linking the bacterial community in pea aphids with host-plant use and natural enemy resistance. Ecol. Entomol..

[11] Leclercq S., Theze J., Chebbi M.A., Giraud I., Moumen B., Ernenwein L., Greve P., Gilbert C., Cordaux R. (2016). Birth of a W sex chromosome by horizontal transfer of *Wolbachia* bacterial symbiont genome. Proc. Natl. Acad. Sci. USA.

[12] McCutcheon J.P., Boyd B.M., Dale C. (2019). The Life of an Insect Endosymbiont from the Cradle to the Grave. Curr. Biol..

[13] McCutcheon J.P., Moran N.A. (2012). Extreme genome reduction in symbiotic bacteria. Nat. Rev. Microbiol..

[14] Orlovskis Z., Canale M.C., Thole V., Pecher P., Lopes J.R., Hogenhout S.A. (2015). Insect-borne plant pathogenic bacteria: Getting a ride goes beyond physical contact. Curr. Opin. Insect Sci..

[15] Nadarasah G., Stavrinides J. (2011). Insects as alternative hosts for phytopathogenic bacteria. FEMS Microbiol. Rev..

[16] Chatterjee S., Almeida R.P., Lindow S. (2008). Living in two worlds: The plant and insect lifestyles of *Xylella fastidiosa*. Annu. Rev. Phytopathol..

[17] Duron O., Bouchon D., Boutin S., Bellamy L., Zhou L., Engelstadter J., Hurst G.D. (2008). The diversity of reproductive parasites among arthropods: *Wolbachia* do not walk alone. BMC Biol..

[18] Werren J.H., Skinner S.W., Huger A.M. (1986). Male-killing bacteria in a parasitic wasp. Science.

[19] Gherna R.L., Werren J.H., Weisburg W., Cote R., Woese C.R., Mandelco L., Brenner D.J. (1991). *Arsenophonus nasoniae* gen. nov., sp. nov. the causative agent of the son-killer trait in the parasitic wasp *Nasonia vitripennis*. Int. J. Syst. Bacteriol..

[20] Nadal-Jimenez P., Griffin J.S., Davies L., Frost C.L., Marcello M., Hurst G.D.D. (2019). Genetic manipulation allows in vivo tracking of the life cycle of the son-killer symbiont, *Arsenophonus nasoniae*, and reveals patterns of host invasion, tropism and pathology. Environ. Microbiol..

[21] Ferree P.M., Avery A., Azpurua J., Wilkes T., Werren J.H. (2008). A bacterium targets maternally inherited centrosomes to kill males in *Nasonia*. Curr. Biol..

[22] Duron O., Schneppat U.E., Berthomieu A., Goodman S.M., Droz B., Paupy C., Nkoghe J.O., Rahola N., Tortosa P. (2014). Origin, acquisition and diversification of heritable bacterial endosymbionts in louse flies and bat flies. Mol. Ecol..

[23] Novakova E., Hypsa V., Nguyen P., Husnik F., Darby A.C. (2016). Genome sequence of *Candidatus* Arsenophonus lipopteni, the exclusive symbiont of a blood sucking fly *Lipoptena cervi* (Diptera: Hippoboscidae). Stand. Genom. Sci..

[24] Novakova E., Husnik F., Sochova E., Hypsa V. (2015). *Arsenophonus* and *Sodalis* Symbionts in Louse Flies: An Analogy to the *Wigglesworthia* and *Sodalis* System in Tsetse Flies. Appl Environ. Microbiol..

[25] Hosokawa T., Nikoh N., Koga R., Sato M., Tanahashi M., Meng X.Y., Fukatsu T. (2012). Reductive genome evolution, host-symbiont co-speciation and uterine transmission of endosymbiotic bacteria in bat flies. ISME J..

[26] Morse S.F., Bush S.E., Patterson B.D., Dick C.W., Gruwell M.E., Dittmar K. (2013). Evolution, multiple acquisition, and localization of endosymbionts in bat flies (Diptera: Hippoboscoidea: Streblidae and Nycteribiidae). Appl Environ. Microbiol..

[27] Jousselin E., Coeur d’Acier A., Vanlerberghe-Masutti F., Duron O. (2013). Evolution and diversity of *Arsenophonus* endosymbionts in aphids. Mol. Ecol..

[28] Hall A.A., Morrow J.L., Fromont C., Steinbauer M.J., Taylor G.S., Johnson S.N., Cook J.M., Riegler M. (2016). Codivergence of the primary bacterial endosymbiont of psyllids versus host switches and replacement of their secondary bacterial endosymbionts. Environ. Microbiol..

[29] Gottlieb Y., Ghanim M., Gueguen G., Kontsedalov S., Vavre F., Fleury F., Zchori-Fein E. (2008). Inherited intracellular ecosystem: Symbiotic bacteria share bacteriocytes in whiteflies. FASEB J..

[30] Kobialka M., Michalik A., Swierczewski D., Szklarzewicz T. (2020). Complex symbiotic systems of two treehopper species: *Centrotus cornutus* (Linnaeus, 1758) and *Gargara genistae* (Fabricius, 1775) (Hemiptera: Cicadomorpha: Membracoidea: Membracidae). Protoplasma.

[31] Kobialka M., Michalik A., Walczak M., Junkiert L., Szklarzewicz T. (2016). *Sulcia* symbiont of the leafhopper *Macrosteles laevis* (Ribaut, 1927) (Insecta, Hemiptera, Cicadellidae: Deltocephalinae) harbors *Arsenophonus* bacteria. Protoplasma.

[32] Salar P., Sémétey O., Danet J.L., Boudon-Padieu E., Foissac X. (2010). “*Candidatus* Phlomobacter fragariae” and the proteobacterium associated with the low sugar content syndrome of sugar beet are related to bacteria of the arsenophonus clade detected in hemipteran insects. Eur. J. Plant. Pathol..

[33] Bressan A. (2014). Emergence and evolution of *Arsenophonus* bacteria as insect-vectored plant pathogens. Infect. Genet. Evol..

[34] Danet J.L., Foissac X., Zreik L., Salar P., Verdin E., Nourrisseau J.G., Garnier M. (2003). “*Candidatus* Phlomobacter fragariae” Is the Prevalent Agent of Marginal Chlorosis of Strawberry in French Production Fields and Is Transmitted by the Planthopper *Cixius wagneri* (China). Phytopathology.

[35] Nourrisseau J.G., Lansac M., Garnier M. (1993). Marginal chlorosis, a new disease of strawberries associated with a bacteriumlike organism. Plant Dis..

[36] Zreik L., Bove J.M., Garnier M. (1998). Phylogenetic characterization of the bacterium-like organism associated with marginal chlorosis of strawberry and proposition of a *Candidatus* taxon for the organism, ‘*Candidatus* Phlomobacter fragariae’. Int. J. Syst. Bacteriol..

[37] Tanaka M., Nao M., Usugi T. (2006). Occurrence of strawberry marginal chlorosis caused by “*Candidatus* Phlomobacter fragariae” in Japan. J. Gen. Plant Pathol..

[38] Semetey O., Gatineau F., Bressan A., Boudon-Padieu E. (2007). Characterization of a gamma-3 Proteobacteria Responsible for the Syndrome “Basses Richesses” of Sugar Beet Transmitted by *Pentastiridius* sp. (Hemiptera, Cixiidae). Phytopathology.

[39] Gatineau F., Jacob N., Vautrin S., Larrue J., Lherminier J., Richard-Molard M., Boudon-Padieu E. (2002). Association with the Syndrome “Basses Richesses” of Sugar Beet of a *Phytoplasma* and a Bacterium-Like Organism Transmitted by a *Pentastiridius* sp. Phytopathology.

[40] Terlizzi F., Babini A.R., Lanzoni C., Pisi A., Credi R., Foissac X., Salar P. (2007). First report of a γ 3-Proteobacterium associated with diseased strawberries in Italy. Plant Dis..

[41] Salar P., Danet J.L., Pommier J.-J., Foissac X. (2010). The biology of *Cixius wagneri*, the planthopper vector of ‘*Candidatus* Phlomobacter fragariae’ in strawberry production tunnels and its consequence for the epidemiology of strawberry marginal chlorosis. Julius Kühn Arch..

[42] Bressan A., Semetey O., Arneodo J., Lherminier J., Boudon-Padieu E. (2009). Vector transmission of a plant-pathogenic bacterium in the *Arsenophonus* clade sharing ecological traits with facultative insect endosymbionts. Phytopathology.

[43] Novakova E., Hypsa V., Moran N.A. (2009). *Arsenophonus*, an emerging clade of intracellular symbionts with a broad host distribution. BMC Microbiol..

[44] Bressan A., Terlizzi F., Credi R. (2012). Independent origins of vectored plant pathogenic bacteria from arthropod-associated *Arsenophonus* endosymbionts. Microb. Ecol..

[45] Danet J.L., Fimbeau S., Pommier J.-J., Couture C., Foissac X. (2010). Detection of phloem restricted bacteria responsible for strawberry marginal chlorosis (SMC) by real-time PCR in a single assay. Julius Kühn Arch..

[46] Batailler B., Lemaitre T., Vilaine F., Sanchez C., Renard D., Cayla T., Beneteau J., Dinant S. (2012). Soluble and filamentous proteins in *Arabidopsis* sieve elements. Plant Cell Environ..

[47] Faoro F., Tornaghi R. (1982). Effects of simultaneous fixation with glutaraldehyde, picric acid and osmium tetroxide on P-proteins in *Vitis vinifera*. Caryologia.

[48] Ernst A.M., Jekat S.B., Zielonka S., Muller B., Neumann U., Ruping B., Twyman R.M., Krzyzanek V., Prufer D., Noll G.A. (2012). Sieve element occlusion (SEO) genes encode structural phloem proteins involved in wound sealing of the phloem. Proc. Natl. Acad. Sci. USA.

[49] Knoblauch M., Peters W.S., Ehlers K., van Bel A.J. (2001). Reversible calcium-regulated stopcocks in legume sieve tubes. Plant Cell.

[50] Dinant S., Clark A.M., Zhu Y., Vilaine F., Palauqui J.C., Kusiak C., Thompson G.A. (2003). Diversity of the superfamily of phloem lectins (phloem protein 2) in angiosperms. Plant Physiol..

[51] Koh E.J., Zhou L., Williams D.S., Park J., Ding N., Duan Y.P., Kang B.H. (2012). Callose deposition in the phloem plasmodesmata and inhibition of phloem transport in citrus leaves infected with “*Candidatus* Liberibacter asiaticus”. Protoplasma.

[52] Kim J.S., Sagaram U.S., Burns J.K., Li J.L., Wang N. (2009). Response of sweet orange (*Citrus sinensis*) to ‘*Candidatus* Liberibacter asiaticus’ infection: Microscopy and microarray analyses. Phytopathology.

[53] Musetti R., Buxa S.V., De Marco F., Loschi A., Polizzotto R., Kogel K.H., van Bel A.J. (2013). Phytoplasma-triggered Ca(2+) influx is involved in sieve-tube blockage. Mol. Plant Microbe Interact..

[54] Musetti R., Paolacci A., Ciaffi M., Tanzarella O.A., Polizzotto R., Tubaro F., Mizzau M., Ermacora P., Badiani M., Osler R. (2010). Phloem cytochemical modification and gene expression following the recovery of apple plants from apple proliferation disease. Phytopathology.

[55] Faoro F., Tornaghi R. (1983). Cytochemical identification of callose deposits around plasmodesmata of virus-infected phloem cells. Ultramicroscopy.

[56] Faoro F., Tornaghi R., Belli G. (1991). Localization of closteroviruses on grapevine thin sections and their identification by immunogold labelling. J. Phytopathol..

[57] Pastrana A.M., Watson D.C., Gordon T.R. (2019). Transmission of *Fusarium oxysporum* f. sp. fragariae through stolons in strawberry plants. Plant Dis..

[58] Gordon T.R., Kirkpatrick S.C., Shaw D.V., Larson K.D. (2002). Differential infection of mother and runner plant generations by *Verticillium dahliae* in a high elevation strawberry nursery. HortScience.

